# Establishment, Characterization and Chemosensitivity of Three Mismatch Repair Deficient Cell Lines from Sporadic and Inherited Colorectal Carcinomas

**DOI:** 10.1371/journal.pone.0052485

**Published:** 2012-12-31

**Authors:** Claudia Maletzki, Saskia Stier, Ulrike Gruenert, Michael Gock, Christiane Ostwald, Friedrich Prall, Michael Linnebacher

**Affiliations:** 1 Division of Molecular Oncology and Immunotherapy, University of Rostock, Rostock, Germany; 2 Department of General, Thoracic, Vascular and Transplantation Surgery, University of Rostock, Rostock, Germany; 3 Institute of Pathology, University of Rostock, Rostock, Germany; Virginia Commonwealth University, United States of America

## Abstract

**Background:**

Colorectal cancer (CRC) represents a morphologic and molecular heterogenic disease. This heterogeneity substantially impairs drug effectiveness and prognosis. The subtype of mismatch repair deficient (MMR-D) CRCs, accounting for about 15% of all cases, shows particular differential responses up to resistance towards currently approved cytostatic drugs. Pre-clinical *in vitro* models representing molecular features of MMR-D tumors are thus mandatory for identifying biomarkers that finally help to predict responses towards new cytostatic drugs. Here, we describe the successful establishment and characterization of three patient-derived MMR-D cell lines (HROC24, HROC87, and HROC113) along with their corresponding xenografts.

**Methodology:**

MMR-D cell lines (HROC24, HROC87, and HROC113) were established from a total of ten clinicopathological well-defined MMR-D cases (120 CRC cases in total). Cells were comprehensively characterized by phenotype, morphology, growth kinetics, invasiveness, and molecular profile. Additionally, response to clinically relevant chemotherapeutics was examined *in vitro* and *in vivo*.

**Principal Findings:**

Two MMR-D lines showing CIMP-H derived from sporadic CRC (HROC24: K-ras^wt^, B-raf^mut^, HROC87: K-ras^wt^, B-raf^mut^), whereas the HROC113 cell line (K-ras^mut^, B-raf^wt^) was HNPCC-associated. A diploid DNA-status could be verified by flow cytometry and SNP Array analysis. All cell lines were characterized as epithelial (EpCAM^+^) tumor cells, showing surface tumor marker expression (CEACAM^+^). MHC-class II was inducible by Interferon-γ stimulation. Growth kinetics as well as invasive potential was quite heterogeneous between individual lines. Besides, MMR-D cell lines exhibited distinct responsiveness towards chemotherapeutics, even when comparing *in vitro* and *in vivo* sensitivity.

**Conclusions:**

These newly established and well-characterized, low-passage MMR-D cell lines provide a useful tool for future investigations on the biological characteristics of MMR-D CRCs, both of sporadic and hereditary origin. Additionally, matched patient-derived immune cells allow for comparative genetic studies.

## Introduction

Developing preclinical cancer models has substantially contributed to a more detailed understanding of colon carcinoma (CRC) initiation and progression [1], [2]. Pivotal to these studies has been the growing appreciation of the histological and genetical heterogeneity that exists within CRC. At present, at least three major molecular mechanisms, i.e. chromosomal instability, the CpG island methylator phenotype (CIMP) as well as high-degree of microsatellite instability (MSI or MMR-D for mismatch repair deficiency), have been identified as promoters of CRC carcinogenesis [3]–[6].

MMR-D accounts for 15% of all CRCs [5]; with 3% being associated with hereditary non-polyposis colorectal carcinoma (HNPCC), and the remaining 12% arise sporadically. MMR-D results from functional inactivation of DNA mismatch repair (MMR) genes; to the most part MLH1 and MSH2. Irrespective of some genetic and epigenetic differences that exist between these two types, there are several features common to both sporadic and inherited MMR-D tumors. They typically possess strong lymphocytic infiltration, an enhanced tumor cell apoptosis, and a distinct response to adjuvant chemotherapy (i.e. 5-fluoruracil- (5-FU) and cisplatin-based therapies) [7]. Correct prediction of cytotoxic agents’ efficacy is a crucial step to improve the outcome of patients suffering from a MMR-D CRC. To date, the mutational status of K-ras is the most recognized predictive molecular marker in CRC. Additional novel markers have been found to be helpful in identifying patients likely to benefit from (e.g. EGFR-) targeted therapies [8]. These include B-raf^V600E^ mutations and loss of PTEN expression.

Mandatory for identifying accurate novel molecular markers and for testing innovative treatment regimen is the availability of suitable *in vitro* and *in vivo* models. Cell line establishment has been described to be successful either directly from fresh tumor tissue or from so-called xenopatients by engrafting tumor fragments in immunocompromised mice before the *in vitro* culture step. For both cases, maintenance of the original tumor’s cell differentiation, morphology and molecular signature is initially warranted [9]. Although some of the resulting MMR-D CRC cell lines were biologically examined and made commercially available [10], they usually are of high passage and thus do not longer reflect the biology of the original tumor, like growth behaviour, morphology and mutational profile [11], [12]. Therefore, the ongoing development of novel low-passage MMR-D CRC lines is imperative. Ideally, a complete set of cell lines, together with matched xenopatients and autologous immune cells, should be at hand for development of novel therapeutic approaches.

In this study, we describe a feasible and straightforward method for establishing new MMR-D cell lines, along with their corresponding xenopatients. Subsequent detailed analysis of tumor biology, genetic, as well as *in vitro* and *in vivo* chemosensitivity towards selected antineoplastic drugs provides a ready basis for preclinical evaluation of innovative treatment regimens. Such patient-derived cell line sets are especially ideal tools to optimize development of individualized therapeutic strategies in the near future.

## Materials and Methods

### Tumor Preparation, Xenografting & Cell Line Establishment

Primary CRC resection specimens were received fresh from surgery, with informed written patient consent (n = 10). All procedures were approved by the Ethics Committee of the Medical faculty, University of Rostock (Ethikkommission an der Medizinischen Fakultät der Universität Rostock, St.-Georg-Str. 108, 18055 Rostock, Germany; reference number II HV 43/2004) in accordance with generally accepted guidelines for the use of human material. Tumor samples were cut into small pieces. For cryopreservation and subsequent xenografting, pieces (3×3×3 mm) were frozen (FCS, 10% DMSO) at −80°C. Other pieces were stored in liquid nitrogen for molecular analysis. Cell culture was started from single cell suspensions, seeded on collagen-coated plates in Quantum tumor medium (+10% FCS, 2 mM L-glutamine, antibiotics and antimycotics) and incubated at 37°C in a humidified atmosphere of 5% C0_2_. All cell culture reagents were obtained from PAA (Cölbe, Germany), antibiotics and antifungal agents were provided by the university hospital’s pharmacy.

Medium was changed regularly. Initial passage into a 25 cm^2^ culture flask was performed when tumor cell growth was observed. Continually growing cell cultures were further passaged and regularly stocked in low passages.

For *in vivo* engraftment, six-week-old female NMRI nu/nu mice were used as recipients. Mice were bred in the university’s animal facility and maintained in specified pathogen-free conditions. All experimental procedures were carried out in strict accordance with the recommendations in the Guide for the Care and Use of Laboratory Animals of the National Institutes of Health. The protocol was approved by the Committee on the Ethics of Animal Experiments of the University of Rostock (Landesamt für Landwirtschaft, Lebensmittelsicherheit und Fischerei Mecklenburg-Vorpommern; Thierfelder Str. 18, 18059 Rostock, Germany; permit number: LALLF M-V/TSD/7221.3-1.1-071-10). All surgery was performed under Ketamin/Xylazin anesthesia (dose: 90/25 mg/kg bw), and all efforts were made to minimize suffering. Subcutaneous (s.c.) tumor implantation was performed as described [13]. Established xenografts (≥1500 mm^3^) were removed and underwent *in vitro* culture protocols as described above.

### Histology and Immunohistochemistry of Original Tumors

Histopathological examination of primary tumors was done according to standard protocols for clinicopathological CRC staging [14] and additional staging information was compiled from patients’ clinical charts. H & E sections; β-catenin, MLH1, and MSH2 immunostainings were obtained from paraffin-embedded tumors.

### Molecular Analysis

Molecular classification was done according to [3]. These data as well as staging information compiled from the clinical charts are summarized in Tables 1–3. MMR-D was examined using the Bethesda panel and additionally the mononucleotide marker Cat25 [15]. Mutational analyses of the APC, p53, K-Ras and B-Raf^V600E^ genes were done as described. Finally, DNA-methylation in CIMP-sensitive promoters was traced by the MethyLight technology with a modified marker panel originally published by [16]. Chromosomal instability (CIN) was assessed using SNP Array 6.0 from Affymetrix (Cleveland, OH) according to manufacturer’s instructions.

**Table 1:** Clinical and pathological characteristics of patients as well as cell line establishment protocol.

Tumor-ID	Age/Gender	Tumor location	TNM-Stage	Tumor type	Molecular type	β-Catenin translocation	MLH1IHC	Corresponding xenograft	Direct cell line establishment	Cell line from xenograft	Paired B-LCL
HROC24	98/m	colon ascendens	G2T2N0M0	primary adenocarcinoma	spMMR-D	negative	−	+	+	+	yes
HROC29	59/m	colon ascendens	G3T3N2M1	primary adenocarcinoma	HNPCC	negative	−	+	−	−	yes
HROC48	68/m	colon transversum	G3T2N1M0	primary adenocarcinoma	spMMR-D	negative	−	+	−	−	n.d.
HROC50	67/f	colon ascendens	G2T4N0M0	primary adenocarcinoma	spMMR-D	negative	−	+	−	+/−	yes
HROC53	72/f	colon ascendens	G3T3N0M0	primary adenocarcinoma	spMMR-D	positive	−	+	−	−	yes
HROC71	52/m	caecum	G2T3N0M0	primary adenocarcinoma	HNPCC	positive	−	+	−	+/−	yes
HROC87	76/f	colon ascendens	G3T3N0M0	primary adenocarcinoma	spMMR-D	negative	−	+	−	+	yes
HROC108	81/f	colon ascendens	G3T3N0M0	primary adenocarcinoma	spMMR-D	positive	−	+	−	−	n.d.
HROC109	75/m	colon transversum	G3T4N2M0	primary adenocarcinoma	spMMR-D	negative	−	+	−	−	n.d.
HROC113	41/f	colon ascendens	G3T4N2Mx	primary adenocarcinoma	HNPCC	negative	−	+	+	−	yes

m – male, f – female, spMMR-D – sporadic mismatch repair deficient, HNPCC – hereditary non-polyposis colorectal carcinoma, B-LCL – B lymphoid cell line,+– positive, − – negative; IHC – immunohistochemistry; n.d. – not done.

**Table 2 pone-0052485-t002:** Molecular characterization of MMR-D CRC cell lines.

Cell line (HROC.)	Mutation								MMR-D status					
	p53				APC	K-Ras		B-Raf	BAT25	BAT26	CAT26	D5S346	D17S250	D2S123
	ex 5	ex 6	ex 7	ex 8	ex 15	cd 12	cd13	V600E						
**24P**	wt	wt	wt	wt	mut	wt	wt	mut	−5	−5	−8	+18	−6/wt	−6/−4
**87X**	wt	mut	mut	wt	wt	wt	wt	mut	−6	−10/−6	−12	−2/+4	+2/wt	0
**113P**	wt	wt	wt	wt	wt	mut	wt	wt	0	−8	−8/−5	0	−6/wt	0

wt – wildtype, mut – mutated, ex – exon, cd – codon.

**Table 3:** DNA-methylation profile of MMR-D CRC cell lines.

Cell line	DNA-methylation					
	MLH1	CDKN2A	NEUROG1	CRABP1	CACNA1G	MGMT
**HROC24P**	+	+	+	+	+	+
**HROC87X**	+	+	+	+	+	−
**HROC113P**	−	−	−	−	−	+

+ – methylated; − – not methylated.

### Generation of Peripheral B Cell Cultures from Primary Tumors

B-lymphoid cell lines (B-LCLs) were generated from purified peripheral blood leukocytes by Epstein-Barr virus (EBV)-transformation as described [17]. Outgrowing B-LCL cultures were harvested, expanded, characterized, and frozen.

### *In vitro* Growth Kinetics, Ploidy and Cell Cycle Analysis

Population doubling times were determined by viable cells seeded into replicate 25 cm^2^ flasks and daily counted for seven days. Ploidy and cell cycle analysis was conducted by flow cytometry (FACSCalibur; BD Biosciences, Heidelberg, Germany) as described [18]. Human blood leukocytes were used as diploid controls.

### Flow Cytometry and Cytokine Secretion Pattern of Primary Cell Lines

Cell surface marker expression on established tumor cell lines was traced by flow cytometry with and without IFN-γ pre-treatment using a panel of Abs (for details please see Table 4). Samples were analysed using CellQuest software (BD Biosciences). Cytokine release was determined from cell free supernatants, harvested at different time points and quantified by ELISA according to the manufacturer’s instructions.

**Table 4:** Flow cytometric phenotyping of primary MMR-D CRC cell lines & MHC expression with and without IFN-γ pre-treatment (% positive cells).

Antigen		HROC24P	HROC87X	HROC113P
**CD11b**		0.0	0.0	0.0
**CD15**		80.8	53.2	40.7
**CD20**		0.0	0.0	0.0
**CD24**		0.0	0.0	0.0
**CD28**		0.0	0.0	0.0
**CD34**		0.0	0.0	0.0
**CD43**		0.0	0.0	0.0
**CD44**		60.4	49.1	42.9
**CD45**		0.0	0.0	0.0
**CD45ra**		0.0	0.0	0.0
**CD45rb**		7.8	8.3	5.8
**CD45ro**		0.0	0.0	0.0
**CD50**		0.0	0.0	0.0
**CD55**		11.5	15.6	21.2
**CD56**		0.0	0.0	0.0
**CD58**		79.8	11.3	10.8
**CD62L**		0.0	0.0	0.0
**CEACAM**		53.9	12.7	63.4
**CD71**		77.1	67.9	75.0
**CD80**		11.2	56.9	75.3
**EpCAM**		99.5	98.9	99.3
**HLA A2**		0.0	0.0	89.2
**MHC I**	**− IFN-γ**	0.0	1.0	6.8
	**+ IFN-γ**	0.0	1.0	93.8
**MHC II**	**− IFN-γ**	0.0	0.0	0.0
	**+ IFN-γ**	94.2	73.3	95.0

Data are given from one representative experiment out of four replicates.

### Matrigel Invasion Assay

Tumor cells invasiveness was examined using a matrigel-based assay according to [19] with minor modifications. Cells on the lower surface were quantified after 72 hours of incubation by MTT assay (Promega, Mannheim, Germany) and absorbance measurement at 490 nm (reference 620 nm). Data are expressed as percentage invasion versus the highly invasive CRC line HCT116 (unpublished own observation) set to 100%.

### Mycoplasma and Viral Infection

Mycoplasma contamination was tested by the 16S-rRNA-gene-based polymerase chain reaction (PCR) amplification method from cell lysates. Amplification was carried out in a total volume of 25 µl (2 mM MgCl_2_, 0.5 mM each primer (Metabion, Martinsried, Germany). Primers were: forward: 5′-GGC GAA TGG GTG AGT AAC ACG-3′; reverse: 5′-CGG ATA ACG CTT GCG ACC TAT G-3′ yielding an approximately 500 bp product (conditions: 94°C, 5 min, 94°C, 1 min; 60°C, 1 min; 72°C, 90 s; 40 cycles). Potential polyomavirus infection was tested from gDNA according to [20]. PCR for detecting HBV, HCV, or HIV in tumor cells was kindly performed by the Institute of Medical Microbiology, Virology and Hygiene (University of Rostock).

### *In vitro* and *in vivo* Chemosensitivity

Cells were seeded into 96-well microtiter plates (5×10^3^ or 1×10^4^ cells/well). Two days after plating, triplicate wells were treated with increasing drug concentrations (pharmacy of the university hospital Rostock). This procedure was repeated after three days of treatment ( = two cycles). Cellular metabolic activity in treated versus control wells was estimated by MTT assay as described above. Drug effects were determined at the level of 50% inhibition (IC_50_) compared to controls.

Thereafter, response to selected therapeutics was tested *in vivo*. 5×10^6^ cells were injected s. c. into nude mice. Additionally, tumor fragments of HNPCC-derived xenografts (HROC29, HROC71) were implanted s.c. into nude mice under anaesthesia (Ketamin/Xylazin 90/25 mg/kg bw) and allowed to grow until tumor establishment. Mice with established tumors received therapeutic applications of selected drugs (i.p.; 20 mg/kg bw each, n = 6–7 mice per group, twice weekly, six times in total). Tumor-carrying mice receiving PBS (n = 7 per group) served as controls. Tumor growth was controlled regularly and volume was estimated according to the formula: V = width^2^ * length * 0.52. All mice (treatment, control) were sacrificed at day 21 or when they became moribund before the tumor volume reached 2000 mm^3^. Tumors were removed for further histological examinations.

### Statistics

Values are reported as the mean ± SD for *in vitro* data and mean ± SEM for *in vivo* data. Statistics were done on *in vivo* experiments. After proving the assumption of normality, differences between saline and treated animals were determined by using the unpaired Student’s *t*-test. If normality failed, the nonparametric Mann–Whitney *U*-Test was applied. The tests were performed by using Sigma-Stat 3.0 (Jandel Corp, San Rafael, CA). The criterion for significance was set to p<0.05.

## Results

### Clinicopathological Patients’ Characteristics

In this study, ten cases of clinicopathological well-defined MMR-D tumors were collected from a series of 120 CRC cases. Samples were obtained from resection specimens without prior therapy. Clinicopathological patient’s characteristics are summarized in Table 1. Seven samples were classified as sporadic MMR-D tumors and three cases as HNPCC. Except for the two of the latter cases (HROC29, HROC113), no distant metastases were present in any of the MMR-D tumor patients.

### Cell Line Establishment

**Figure 1:**
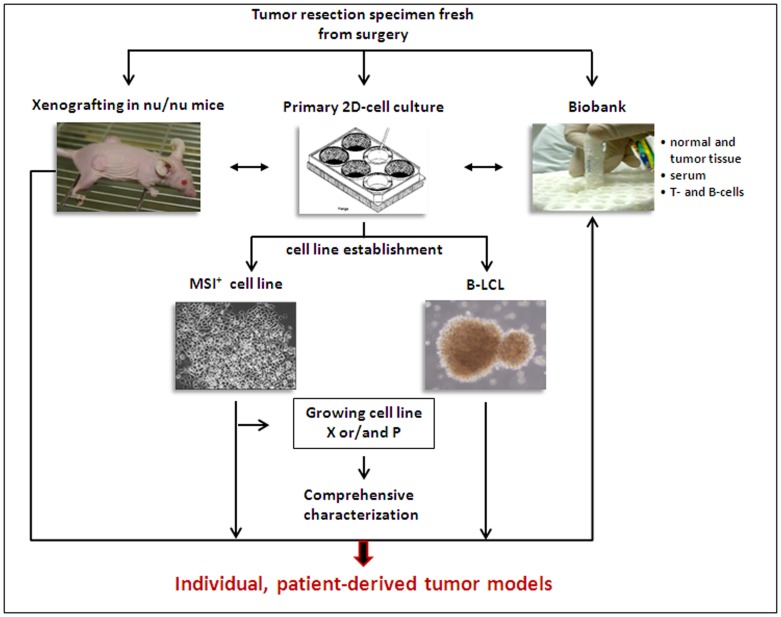
Scheme of the experimental protocol for cell line establishment either fresh from surgery specimens ( = patient-derived cell line) of following xenografting in nu/nu mice ( = xenopatient-derived cell line). Established cell lines were comprehensively characterized and routinely cyropreserved together with xenograft-tissue, immune cells, serum, and normal as well as primary tumor tissue in a biobank. This procedure leads to generation of individualized, patient-derived tumor models available for functional analyses.

To increase the success rate of tumor cell line establishment, *in vitro* and *in vivo* approaches were combined: parts of surgical tumor specimens were either directly processed for *in vitro* cell line establishment or frozen native for subsequent xenopatient generation (Figure 1) [21].

With this method, direct cell line establishment was successful for 2/8 cases. The cell line HROC24P (P = direct cell line establishment from patient) originated from a sporadic CRC patient and HROC113P was derived from a HNPCC patient with a germline MLH1 mutation.

**Figure 2:**
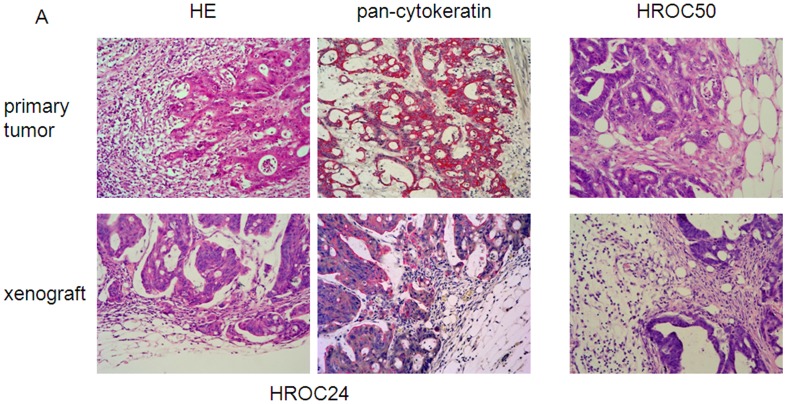
Morphology of primary MMR-D tumors and their corresponding xenografts. HE-histology representing maintenance of HROC24 tumor morphology following xenografting. The pan-cytokeratin staining is strongly positive, consistent with the tumors’ epithelial origin (left upper and lower panel). HE-histology of HROC50 tumors (right upper and lower panel).

In a parallel series of experiments, xenografting was performed on 8/10 tumors by subcutaneous implantation into immunocompromised recipients. Preservation of morphology was confirmed by comparing histology of xenografts with the original tumor (Figure 2).

Tumor growth was obtained in 62% and thus to a significantly higher efficiency than the direct *in vitro* approach (Table 1). However, subsequent cell line establishment was so far only successful in two cases, namely HROC24X and HROC87X (X = xenopatient-derived). For these two lines, continuous *in vitro* growth was observed immediately after starting cultures.

Finally, detailed characterization was performed on the patient-derived cell lines, HROC24P and HROC113P, as well as the xenopatient-derived line HROC87X. As determined by PCR, all MMR-D lines were found free of contaminating mycoplasma or human pathogenic viruses (SV40, JC/BK, HBV, HCV, and HIV; data not shown).

### Molecular Characterization

Comprehensive molecular classification on freshly established MMR-D cell lines was paralleled by examinations on original tumor material as well as on corresponding xenografts (HROC24 and HROC87). This analysis revealed no difference in any of the samples and hence data presented in Table 2 and 3 refer to the cell lines only.

**Figure 3:**
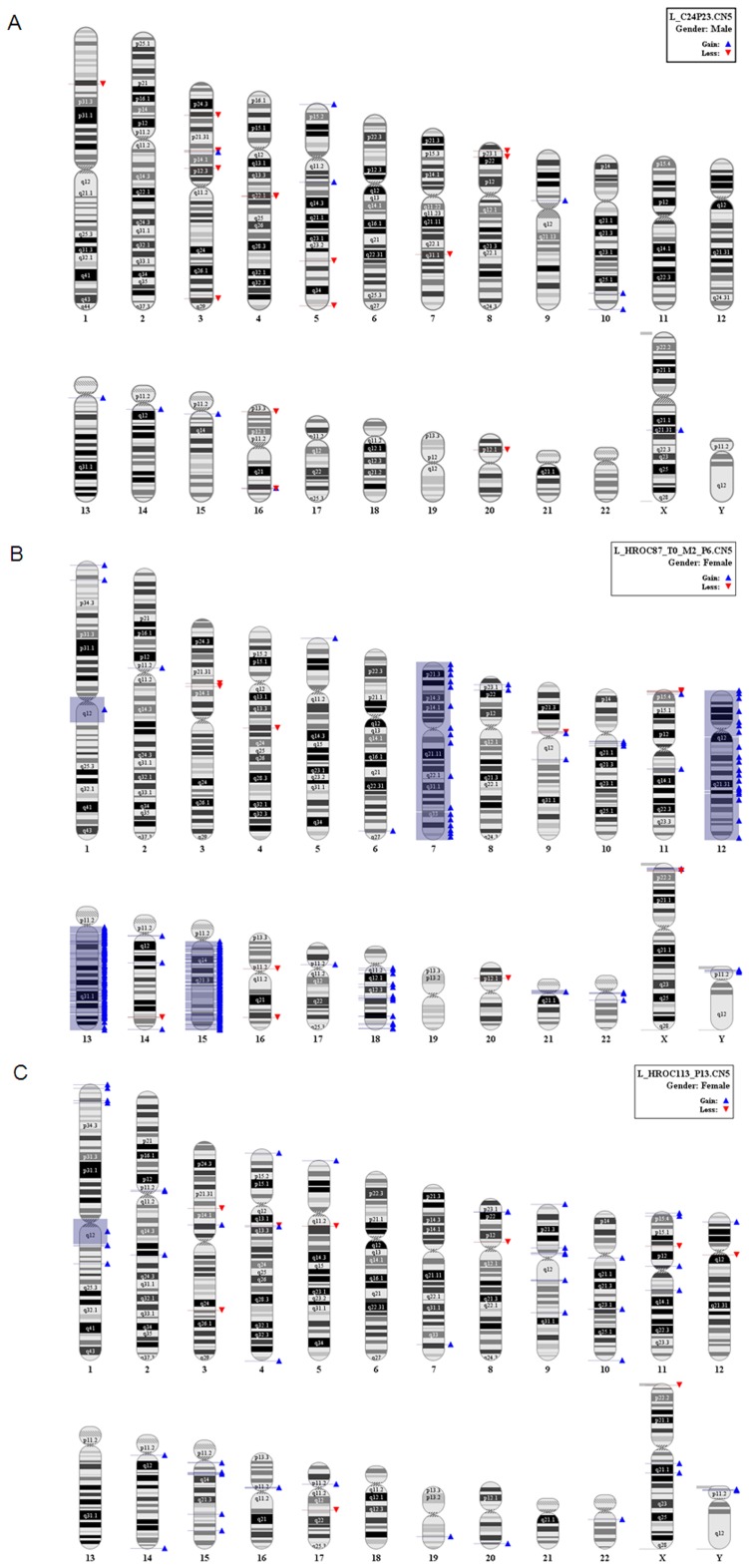
SNP Array 6.0 for assessment of CIN in MMR-D cell lines. Analysis was performed according to manufacturer’s instructions. (A) HROC24P cells, (B) HROC87X cells, and (C) HROC113P cells.

The MMR-D status was evidenced by using the advanced Bethesda panel. All three cell lines exhibited instability in the six markers analyzed. Mutations in tumor-associated genes varied among cell lines. HROC24P and HROC87X displayed high-degree of CIMP (methylation in 6/6 markers), including MLH1 promoter methylation. The HROC113P cell line showed characteristic molecular features associated with the HNPCC syndrome like mutations in codon 12 of the K-ras gene, but wildtype B-raf. Moreover, methylation –except for MGMT– was absent. MMR-D tumors are by definition devoid of or have very low levels of CIN [3]. However, for this detailed characterization, we performed a genomic analysis with very high resolution taking advantage of the SNP Array 6.0. Even in this analysis, very low if any instability on the chromosomal level could be detected with the exception of HROC87X cells, which had an amplification of 13q and 15q (Figure 3A). HROC24P and HROC113P were close to normal (Figure 3B, C).

### Cell Morphology & Phenotyping

**Figure 4:**
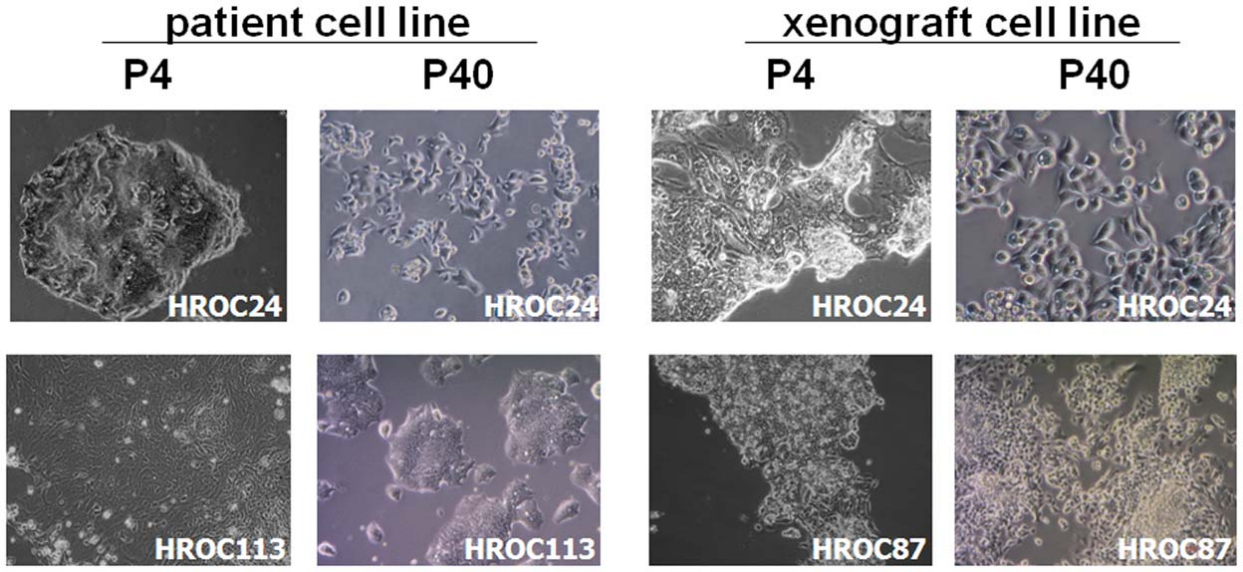
Light microscopy of MMR-D CRC cell lines both directly after establishment (P4) and following long-term *in vitro* culture (P40). (A) Morphology of patient-derived cell lines HROC24P and HROC113P. (B) Morphology of HROC24X and HROC87X. Both cell lines were established from xenopatients as described in material & methods. Original magnification x100.

Determining morphology revealed tight adherence to the bottom of the cell culture flasks. All cell lines were characterized as epithelial-like cells without contaminating fibroblasts. Initially, HROC24P cells proliferated as tightly packed multi-cellular islands (Figure 4 upper panel). Following serial passages, they changed their morphology and appeared as rather undifferentiated small, polygonal and round cells not strictly growing in monolayer (Figure 4 upper panel). Morphology at later passages was identical to HROC24X, which had been generated from a xenopatient (Figure 4, upper panel). HROC87X cells displayed comparable growth behaviour, forming small floating aggregates or grape-like cell clusters (Figure 4 lower panel) with no change in phenotype during long-term culture (≥40 passages).

HROC113P cells strictly grew as monolayers. In the initial cell culture (<4 passages), two different cellular clones were observed. After serial passages, numbers of large cells gradually decreased and were entirely replaced by the dominant smaller cell clone (Figure 4 lower panel). Unlike the other cell lines, they did not grow to complete confluence.

The epithelial phenotype was confirmed by positive immunoreactivity for the epithelial cell adhesion molecule (EpCAM; each >98%; Table 4) as well as expression of the tumor marker CEACAM (Table 3). Further characterisation revealed comparable expression levels for the cellular adhesion and migration marker CD44 and the transferrin receptor CD71, whereas heterogeneous expression of adhesion markers was observed (CD15, CD58). High MHC class I expression was only observed in HROC113P cells, which were additionally found to be HLA-A2 positive (Table 4). MHC class II expression was absent on all cells, but could –to varying degrees– be induced by IFN-γ pre-treatment (Table 4).

### Growth Kinetics, Ploidy & Cellular Invasiveness

To get an idea on the *in vivo* growth behavior of the original tumor, we examined growth kinetic and invasive potential of the cell lines.

**Figure 5:**
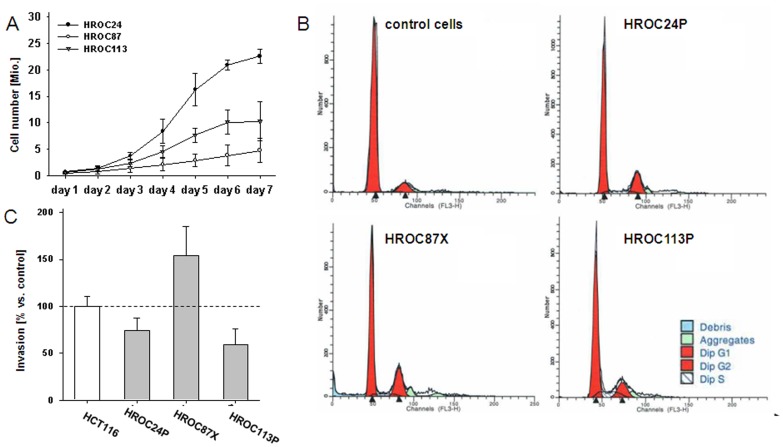
*In vitro* growth kinetic, ploidy analysis & invasiveness of MMR-D tumor cells. (A) Growth curve of HROC24P, HROC87X and HROC113P cells in culture. The results shown are the mean population doubling times ± standard deviation. Results were calculated from three independent assays each performed in duplicates. (B) Exemplary DNA histograms of MMR-D tumor cells compared to normal cells (PBMC). All cells were classified as diploid. (C) Tumor invasiveness was analysed using a matrigel-based assay. Quantification of cellular invasiveness was estimated by MTT assay. Data are expressed as percentage invasion versus HCT116 cells ( = internal positive control). All experiments were repeated at least three times.

As anticipated, growth kinetics were quite different between cells, with HROC24P growing more rapidly than HROC87X and HROC113P cells (33 vs. 50 and 41 hours; Figure 5A). In subsequent cell cycle analysis, no statistical differences between the three cell lines were found in terms of cell numbers in the G0/G1, S, and G2/M phases. In line with the SNP Array 6.0 data, a diploid DNA-status could be verified, too (Figure 5B).

Apart from these findings, we observed considerable differences with regard to the invasive potential (Figure 5C). Highest invasiveness was found for HROC87X cells, which was above the control cell line HCT116. Contrary, the cell lines HROC24P and HROC113P were markedly less invasive.

### Cytokine Secretion Pattern of MMR-D Tumor Cell Lines

Examining Th1 and Th2 cytokine secretion for several days revealed a comparable pattern between cell lines (data not shown). Highest levels were observed for the neutrophil-attracting chemokine IL8. Values of the Th2 cytokine IL4 were identical between all three lines, ranging from 110 to 230 pg/ml (HROC24P vs. HROC113P cells, 4d culture). A similar pattern was seen for IL10. IL6 production was quite low in the MMR-D lines and none of them secreted detectable levels of the immunostimulatory IFN-γ.

### *In vitro* Drug Response

As a first step towards establishing test systems that may predict MMR-D chemosensitivity, an *in vitro* system was used. Exponentially growing cells were treated for a total of six days. In order to mimic the *in vivo* situation, cells received two chemotherapeutic cycles and IC_50_ levels were calculated (Table 5).

**Table 5:** IC_50_ values of antitumor drugs evaluated for MMR-D cell lines.

Cell line	IC_50_ value				
	irinotecan [µM]	cisplatin [µg/ml]	5-FU [µg/ml]	paclitaxel [µM]	gemcitabine [µg/ml]
**HROC24P**	2.2	2.0	0.2	<0.01	0.00015
**HROC87X**	0.9	1.7	0.8	<0.01	0.002
**HROC113P**	12.8	1.3	1.0	2.8	0.00018
**plasma levels (pharmacokinetic)**	10.0	2.0	20.0	50.0–70.0	6.0

Values are given as mean, resulting from at least three independent experiments each performed in triplicates.

These experiments revealed heterogeneous drug responses. In detail, the two sporadic MMR-D cell lines (HROC24P, HROC87X) were sensitive towards irinotecan-mediated growth inhibition; with doses comparable to or even lower than plasma concentrations in patients (Table 5). Interestingly, HROC113P cells did not response to this compound. Comparable results were obtained for 5-FU, with HROC24P being the most sensitive cell line. Again, HROC113P cells showed relative resistance towards 5-FU. All cell lines were susceptible to cisplatin-induced growth arrest.

Thereafter, sensitivity towards several additional cytostatic drugs was tested. The microtubule-stabilizing compound paclitaxel showed effectiveness comparable to the standard drugs. Again, highest efficacy was observed against HROC24P and HROC87X cells, while it was less potent towards HROC113P cells. A somewhat unexpected finding was the high responsiveness towards gemcitabine. All three cell lines showed substantial response, even at very low doses. Inhibition of cell proliferation was achieved at concentrations well below plasma levels under standard therapy.

In summary, *in vitro* chemosensitivity patterns to cytotoxic drugs were very individual with the HNPCC-derived HROC113 tending to be more resistant than their sporadic counterparts.

### *In vivo* Tumorigeneicity & Drug Response

All three cell lines engrafted well and gave rise to growth *in vivo*. Similar to the *in vitro* growth behavior, HROC24P tumors displayed fastest growth, HROC87X tumors grew slowest and HROC113P tumors showed an intermediate growth rate. No distant metastases were detected neither at necropsy, nor following histologic examination of the inner organs (data not shown).

**Figure 6:**
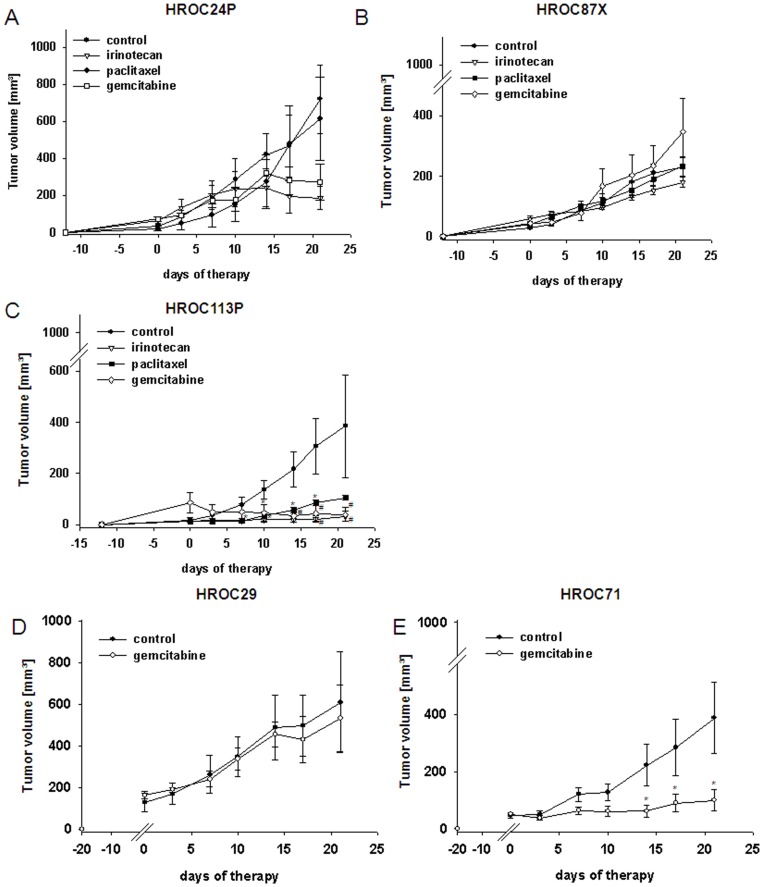
*In vivo* tumorigeneicity & response to selected chemotherapeutics. NMRI nu/nu mice either received s.c. injections of established MMR-D cell lines (5 Mio. cells/mouse; A–C) or were s.c grafted with HROC29 and HROC71 tumor fragments (D, E). For analyzing *in vivo* drug response, mice with established (A) HROC24, (B) HROC87 or (C) HROC113 tumors were treated with irinotecan, paclitaxel or gemcitabine (i.p.; 20 mg/kg bw each, n = 6−7 mice per group). HROC71 and HROC29 xenografts were given gemcitabine (i.p.; 20 mg/kg bw, n = 5−6 mice per group). Therapeutic regimens consisted of six injections in total, applied twice a week. Control animals received equivalent volumes of saline (n = 6−7). Values of are given as mean ± SEM. *p<0.05 vs. control, *U*-Test; ^#^p<0.05 vs. control, *t*-test.

Subsequently, the effectiveness of chemotherapeutics was studied *in vivo* (Figure 6). The topoisomerase-1 inhibitor irinotecan mediated substantial tumor growth control. Unexpected from the *in vitro* results, most pronounced effects were obtained for HROC113P with more than 90% growth inhibition compared to untreated mice. Tumors immediately stopped growing after the first therapy cycle. This was evident until the end of experiments at day 21 (Figure 6C). HROC24P and HROC87X tumors showed a weaker, though still significant response to irinotecan. Tumors tended to keep growing very slowly, finally resulting in sizes less than half of untreated tumors (Figure 6A, B).

Quite the opposite results were obtained for paclitaxel. Despite effective inhibition *in vitro*, this drug affected tumor growth only in the HNPCC-associated HROC113P tumors (Figure 6C) while it had no or only marginal effect on HROC87X and HROC24P tumor growth, respectively (Figure 6A, B).

Due to the high sensitivity of all MMR-D cell lines towards gemcitabine *in vitro*, this drug was consequently applied therapeutically *in vivo*. Most prominent antitumoral effects were observed for HROC113P-tumors, finally leading to complete remission of 4/7 tumors (Figure 6C). HROC24P-tumor growth was also effectively controlled (Figure 6A). HROC87X-tumors behaved completely different (Figure 6B). Here, the tumor growth rate was slightly enhanced by gemcitabine. These results let us hypothesize that HNPCC tumors might probably better respond to this regimen. Consequently, we included two further HNPCC-associated cases, i.e. HROC29 and HROC71. Gemcitabine effectively controlled HROC71 xenograft growth, but failed to affect HROC29 tumors (Figure 6D, E).

## Discussion

Advancement in molecular understanding of CRC biology has emerged important for preclinical and early clinical studies. With regard to MMR-D tumors, differential response up to chemoresistance was observed, yet these findings have so far not ended in personalized treatment regimen [22]. This is, at least in part, attributable to the paucity of well-characterized preclinical models. Establishing novel low-passage MMR-D CRC lines and xenopatients, which provide a virtually unlimited source of tumor material [23], [24], [25], may thus help to improve preclinical optimization of personalized therapy.

In this study, we picked up the idea of establishing and characterizing patient-derived individual MMR-D tumor models for testing treatment modalities. Firstly, to ameliorate the success rate for CRC cell line establishment, an experimental strategy –based on the combination of *in vitro* and *in vivo* approaches– was elaborated. From ten MMR-D tumors included in this study, cell line establishment was successful in three cases: Two cell lines (HROC24 and HROC113) were directly established from surgical resection specimens, whereas one cell line (HROC87) was obtained subsequent to xenografting. In addition, a paired xenograft cell line from the HROC24 tumor was obtained, too. By combining xenografting and direct *in vitro* cell culture, even more individual patient-derived cell lines, reflecting the original tumors’ molecular signature, will probably become available in the future. Moreover, preselection based on criteria like node infiltration, advanced stage, and elevated CEA in serum may increase the rate of successful tumor engraftment rate into immunocompromised mice [25].

The three newly established MMR-D cell lines exhibited variations in terms of morphology and growth kinetic. Confirming their origin, they were recognized as epithelial tumor cells (EpCAM^+^, CEACAM^+^) with heterogeneous adhesion (CD15, CD44, CD58) and co-stimulatory marker (CD80) expression as well as different cytokine secretion patterns. MHC class I was only expressed by HROC113 cells. Whereas MHC-class II expression was normally absent but inducible by Interferon-γ stimulation. These findings are of interest for development of immune-based therapeutic concepts. Of note, paired immune cells, i.e. antigen-presenting B-LCL and (effector) T cells, offer the chance to work in complete autologous settings. For the MMR-D models described here, limited amounts of immune cells are available to us.

Comparing molecular profiles of MMR-D cell lines with original tumor material as well as matched xenopatients revealed virtually no differences. Somehow unexpected, tumor-associated mutations, MMR-D-status, DNA-methylation in CIMP-specific promoters and LOH profiles remained identical between these samples. It is noteworthy that cells were exclusively studied at early *in vitro* passages (<50), thereby reflecting the original tumors’ biology and not a genotypic and phenotypic heterogeneity that could arise following long-term culture [26]. The molecular signatures found in the MMR-D cell lines corresponded well with the pathways of carcinogenesis ascribed to them. These include B-raf^V600E^ mutations and high-degree promoter methylations in CpG islands of sporadic tumors (HROC24P, HROC87X); and a K-ras mutation but CIMP^neg^ in the HNPCC-associated HROC113P. Which molecular markers predict patients’ clinicopathological outcome best is currently a matter of debate. Bae and colleagues recently claimed that CIMP status might be crucial for MMR-D CRCs response to cytotoxic chemotherapeutics [27]. They correlated clinicopathological features with CIMP status and observed that patients with high level of methylation had worse clinical outcome than their CIMP^neg^ counterparts. Also, BRAF^V600E^ mutations, which have never been found in CIMP^neg^ tumors, seem to be another independent prognosticator of poor outcome and thus an important confounder to the prognostic role of CIMP [28], [29]. These findings further endorse the importance of developing subtype-specific therapeutic strategies.

Additionally, it led us to hypothesize that even molecular closely matched cancer cell lines may respond different towards certain chemotherapeutics. Indeed, chemosensitivity varied between our cell lines.MMR-D. Of particular interest was the difference in terms of *in vitro* and *in vivo* response, especially of the HNPCC-associated HROC113P cells. MMR-DD tumors were described to be more sensitive towards microtubule-stabilizing agents than their CIN counterparts [22], [29], [30]. MMR-DMMR-DIn our study, paclitaxel proved effective *in vitro*, but failed *in vivo*.

Of particular interest was the sensitivity towards gemcitabine, the standard drug for treatment of pancreatic carcinoma – but not of CRC [31]. 4/7 HROC113 xenopatients were cured with this drug. Clinical studies showed controversial effects of gemcitabine either alone or in combination with other anti-neoplastic agents on CRC patients [32]–[34]. These observations fit well with our results. Effective growth inhibition was observed in two cases (HROC24, HROC71) and ineffectiveness in two others (HROC29, HROC87). Response to gemcitabine seems thus quite individual. This should, however, be tested on larger patient cohorts in order to get more reliable information. Additionally, experiments may be performed in nude rats, which were found to resemble humans in terms of metabolism and drug pharmacokinetic profiles more accurately than mice [25], [35]. Limitations of our experimental approach may be found in the comparably long time for obtaining tumor models before testing responsiveness and/or drug resistance. Particularly when facing metastases, drugs that proved to be effective against the primary tumor often fail to affect metastatic lesions. To expand the research in the future –especially for better preclinical testing of approved and new therapies– short-term primary cultures preserving to some extent the three-dimensional tumor organization may be used instead [37].

These experiments shall be combined with systems biology-based approaches for predicting patient outcome and responsiveness to conventional and novel, targeted treatment strategies [36]. Based on mathematically predicted system models, responses can be validated *in vitro* and subsequently verified in xenopatients. This will help to identify patients likely to benefit from optimal care, while sparing those unlikely to benefit. Thus, unnecessary toxicity and costs are avoided.

Our comprehensive biobanking strategy for CRC includes the collection of primary material (frozen and paraffin-embedded tumor and normal tissue as well as lymphocytes) together with established xenografts, patient as well as xenopatient-derived cell lines and matched B-LCLs. Recently, we broadened this approach by collecting tumor samples and correlating clinical data from multiple clinical centers in the context of a third-party funded biobanking initiative (http://www.northgermantumorbank-crc.de).

This strategy may thus be ideal to address the question of whether cell lines, xenopatients or both are suited models for predicting treatment and response. If so, this strategy will pave the way towards truly personalized therapy.

## References

[1] Hewitt RE, McMarlin A, Kleiner D, Wersto R, Martin P, et al (2000) Validation of a model of colon cancer progression. J Pathol 192: 446–454. 11113861 10.1002/1096-9896(2000)9999:9999<::AID-PATH775>3.0.CO;2-K

[2] Voskoglou-Nomikos T, Pater JL, Seymour L (2003) Clinical predictive value of the in vitro cell line, human xenograft, and mouse allograft preclinical cancer models. Clin Cancer Res 9: 4227–4239. Review. 14519650

[3] Ostwald C, Linnebacher M, Weirich V, Prall F (2009) Chromosomally and microsatellite stable colorectal carcinomas without the CpG island methylator phenotype in a molecular classification. Int J Oncol 35: 321–327. 19578746

[4] Harrison S, Benziger H (2011) The molecular biology of colorectal carcinoma and its implications: a review. Surgeon 9: 200–210. Review. 10.1016/j.surge.2011.01.011 21672660

[5] Pritchard CC, Grady WM (2011) Colorectal cancer molecular biology moves into clinical practice. Gut 60: 116–129. Review. 10.1136/gut.2009.206250 PMC3006043 20921207

[6] Kang GH (2011) Four molecular subtypes of colorectal cancer and their precursor lesions. Arch Pathol Lab Med 135: 698–703. 21631262 10.5858/2010-0523-RA.1

[7] Kim ST, Lee J, Park SH, Park JO, Lim HY, et al (2010) Clinical impact of microsatellite instability in colon cancer following adjuvant FOLFOX therapy. Cancer Chemother Pharmacol 66: 659–667. 20033812 10.1007/s00280-009-1206-3

[8] Bass A (2011) Impact of KRAS and BRAF gene mutations on targeted therapies in colorectal cancer. J Clin Oncol 29: 2728–2729. 21646605 10.1200/JCO.2011.36.1816

[9] Marangoni E, Vincent-Salomon A, Auger N, Degeorges A, Assayag F, et al (2007) A new model of patient tumor-derived breast cancer xenografts for preclinical assays. Clin Cancer Res 13: 3989–3998. 17606733 10.1158/1078-0432.CCR-07-0078

[10] Ku JL, Shin YK, Kim DW, Kim KH, Choi JS, et al (2010) Establishment and characterization of 13 human colorectal carcinoma cell lines: mutations of genes and expressions of drug-sensitivity genes and cancer stem cell markers. Carcinogenesis 31: 1003–1009. 20176655 10.1093/carcin/bgq043

[11] Danes BS, Deangelis P, Traganos F, Melamed MR, Alm T (1988) Demonstration of altered cellular DNA content distribution in long-term colon epithelial cell lines with colon cancer genotypes. Scand J Gastroenterol 23: 840–846. 3227300 10.3109/00365528809090770

[12] Bocsi J, Zalatnai A (1999) Establishment and long-term xenografting of human pancreatic carcinomas in immunosuppressed mice: changes and stability in morphology, DNA ploidy and proliferation activity. J Cancer Res Clin Oncol 125: 9–19. 10037272 10.1007/s004320050236 PMC12199854

[13] Poupon MF, Arvelo F, Goguel AF, Bourgeois Y, Jacrot M, et al (1993) Response of small-cell lung cancer xenografts to chemotherapy: multidrug resistance and direct clinical correlates. J Natl Cancer Inst 85: 2023–2029. 7902445 10.1093/jnci/85.24.2023

[14] Fielding LP, Arsenault PA, Chapuis PH, Dent O, Gathright B, et al (1991) Clinocpatholgoical staging for colorectal cancer: an International Documentation System (IDS) and an Interantional Comprehensive Anatomical Terminology (ICAT). J Gastroenterol Hepatol 6: 325–344. 1912440 10.1111/j.1440-1746.1991.tb00867.x

[15] Findeisen P, Kloor M, Merx S, Sutter C, Woerner SM, et al (2005) T25 repeat in the 3′ untranslated region of the CASP2 gene: a sensitive and specific marker for microsatellite instability in colorectal cancer. Cancer Res 65: 8072–8078. 16166278 10.1158/0008-5472.CAN-04-4146

[16] Ogino S, Nosho K, Kirkner GJ, Kawasaki T, Meyerhardt JA, et al (2009) CpG island methylator phenotype, microsatellite instability, BRAF mutation and clinical outcome in colon cancer. Gut 58: 90–96. 18832519 10.1136/gut.2008.155473 PMC2679586

[17] Klier U, Maletzki C, Klar E, Linnebacher M (2010) Generation of highly pure fusions of colorectal carcinoma and antigen-presenting cells. Langenbecks Arch Surg 395: 365–371. 20309577 10.1007/s00423-010-0598-1

[18] Jaster R, Lichte P, Fitzner B, Brock P, Glass A, et al (2005) Peroxisome proliferator-activated receptor gamma overexpression inhibits pro-fibrogenic activities of immortalised rat pancreatic stellate cells. J Cell Mol Med 9: 670–682. 16202214 10.1111/j.1582-4934.2005.tb00497.x PMC6741639

[19] Ramer R, Hinz B (2008) Inhibition of cancer cell invasion by cannabinoids via increased expression of tissue inhibitor of matrix metalloproteinases-1. J Natl Cancer Inst 100: 59–69. 18159069 10.1093/jnci/djm268

[20] Tognon M, Casalone R, Martini F, De Mattei M, Granata P, et al (1996) Large T antigen coding sequences of two DNA tumor viruses, BK and SV40, and nonrandom chromosome changes in two glioblastoma cell lines. Cancer Genet Cytogenet 90: 17–23. 8780741 10.1016/0165-4608(96)00067-2

[21] Linnebacher M, Maletzki C, Ostwald C, Klier U, Krohn M, et al (2010) Cryopreservation of human colorectal carcinomas prior to xenografting. BMC Cancer 10: 362. 20615215 10.1186/1471-2407-10-362 PMC2910693

[22] Hewish M, Lord CJ, Martin SA, Cunningham D, Ashworth A (2010) Mismatch repair deficient colorectal cancer in the era of personalized treatment. Nat Rev Clin Oncol 7: 197–208. 20177404 10.1038/nrclinonc.2010.18

[23] Kirkland SC, Bailey IG (1986) Establishment and characterisation of six human colorectal adenocarcinoma cell lines. Br J Cancer 53: 79–85. 10.1038/bjc.1986.132 PMC2001395 3718830

[24] Dangles-Marie V, Pocard M, Richon S, Weiswald LB, Assayag F, et al (2007) Establishment of human colon cancer cell lines from fresh tumors versus xenografts: comparison of success rate and cell line features. Cancer Res 67: 398–407. 17210723 10.1158/0008-5472.CAN-06-0594

[25] Julien S, Merino-Trigo A, Lacroix L, Pocard M, Goere D, et al (2012) Characterization of a large panel of patient-derived tumor xenografts representing the clinical heterogeneity of human colorectal cancer. Clin Cancer Res 18: 5314–28. 22825584 10.1158/1078-0432.CCR-12-0372

[26] Gagos S, Iliopoulos D, Tseleni-Balafouta S, Agapitos M, Antachopoulos C, et al (1996) Cell senescence and a mechanism of clonal evolution leading to continuous cell proliferation, loss of heterozygosity, and tumor heterogeneity: studies on two immortal colon cancer cell lines. Cancer Genet Cytogenet 90: 157–165. 8830727 10.1016/s0165-4608(96)00049-0

[27] Bae JM, Kim MJ, Kim JH, Koh JM, Cho NY, et al (2011) Differential clinicopathological features in microsatellite instability-positive colorectal cancers depending on CIMP status. Virchows Arch 459: 55–63. 21494758 10.1007/s00428-011-1080-3

[28] El-Osta H, Falchook G, Tsimberidou A, Hong D, Naing A, et al (2011) BRAF Mutations in Advanced Cancers: Clinical Characteristics and Outcomes. PLoS One 6: 25806. 10.1371/journal.pone.0025806 PMC3198456 22039425

[29] Ogino S, Shima K, Meyerhardt J, McCleary N, Ng K, et al (2012) Predictive and Prognostic Roles of BRAF Mutation in Stage III Colon Cancer: Results from Intergroup Trial CALGB 89803. Clin Cancer Res 18: 890–900. 22147942 10.1158/1078-0432.CCR-11-2246 PMC3271172

[30] Swanton C, Caldas C (2009) Molecular classification of solid tumors: towards pathway-driven therapeutics. Br J Cancer 100: 1517–22. 19367275 10.1038/sj.bjc.6605031 PMC2696770

[31] Castellanos E, Berlin J, Cardin DB (2011) Current treatment options for pancreatic carcinoma. Curr Oncol Rep 13: 195–205. Review. 10.1007/s11912-011-0164-1 21491194

[32] Madajewicz S, Waterhouse DM, Ritch PS, Khan MQ, Higby DJ, et al (2012) Multicenter, randomized phase II trial of bevacizumab plus folinic acid, fluorouracil, gemcitabine (FFG) versus bevacizumab plus folinic acid, fluorouracil, oxaliplatin (FOLFOX4) as first-line therapy for patients with advanced colorectal cancer. Invest New Drugs 30: 772–778. 21120580 10.1007/s10637-010-9598-9

[33] Correale P, Botta C, Cusi MG, Del Vecchio MT, De Santi MM, et al (2012) Cetuximab +/− chemotherapy enhances dendritic cell-mediated phagocytosis of colon cancer cells and ignites a highly efficient colon cancer antigen-specific cytotoxic T-cell response in vitro. Int J Cancer 130: 1577–1589. 21618510 10.1002/ijc.26181

[34] Saif MW, Kaley K, Penney R, Hotchkiss S, Syrigos KN, et al (2011) The efficacy of gemcitabine as salvage treatment in patients with refractory advanced colorectal cancer (CRC): a single institution experience. Anticancer Res 31: 2971–2974. 21868546

[35] O’Reilly T, McSheehy PM, Kawai R, Kretz O, McMahon L, et al (2010) Comparative pharmacokinetics of RAD001 (everolimus) in normal and tumor-bearing rodents. Cancer Chemother Pharmacol 65: 625–39. 19784839 10.1007/s00280-009-1068-8

[36] Hector S, Rehm M, Schmid J, Kehoe J, McCawley N, et al (2012) Clinical application of a systems model of apoptosis execution for the prediction of colorectal cancer therapy responses and personalisation of therapy. Gut 61: 725–733. 22082587 10.1136/gutjnl-2011-300433

[37] Baguley BC, Marshall ES, Finlay GJ (1999) Short-term cultures of clinical tumor material: potential contributions to oncology research. Oncol Res 11: 115–124. 10527071

